# Correlations between Polyacetylene Concentrations in Carrot (*Daucus carota* L.) and Various Soil Parameters

**DOI:** 10.3390/foods5030060

**Published:** 2016-08-30

**Authors:** Lars Kjellenberg, Eva Johansson, Karl-Erik Gustavsson, Artur Granstedt, Marie E. Olsson

**Affiliations:** 1 Department of Plant Breeding, Swedish University of Agriculture, P.O. Box 101, Alnarp 230 53, Sweden; eva.johansson@slu.se (E.J.); karl-erik.gustavsson@slu.se (K.-E.G.); marie.olsson@slu.se (M.E.O.); 2 Biodynamic Research Institute, Skilleby, Järna 153 91, Sweden; arturgranstedt@jdb.se

**Keywords:** carrot, *Daucus carota*, polyacetylenes, falcarinol, falcarindiol, falcarindiol-3-acetate, organic agriculture, soluble sugar, soil parameters

## Abstract

This study assessed the concentrations of three falcarinol-type polyacetylenes (falcarinol, falcarindiol, falcarindiol-3-acetate) in carrots and the correlations between these and different soil traits. A total of 144 carrot samples, from three different harvests taken a single season, were analysed in terms of their polyacetylene concentrations and root development. On one of the harvesting occasions, 48 soil samples were also taken and analysed. The chemical composition of the soil was found to influence the concentrations of falcarinol-type polyacetylenes in carrots. When the total soil potassium level was 200 mg/100 g soil, the concentration of falcarindiol (FaDOH) in the carrot samples was 630 μg/g DW, but when carrots were grown in soil with a total potassium level of 300 mg/100 g soil, the FaDOH concentration in the carrots fell to 445 μg/g DW. Carrots grown in soils generally low in available phosphorus exhibited higher levels of falcarindiol if the soil was also low in available magnesium and calcium. The concentrations of polyacetylenes in carrots were positively correlated with total soil phosphorus level, but negatively correlated with total soil potassium level. Of the three polyacetylenes analysed, FaDOH concentrations were influenced most by changes in soil chemical composition.

## 1. Introduction

Three falcarinol-type C17-polyacetylenes (FaTP) found in carrots have been reported to have both positive and negative traits for human consumption [1,2]. For example, falcarinol (FaOH) has been shown to exert cytotoxic activity against human tumour cells [3] and to stimulate differentiation of mammalian cells at concentrations down to 1 ng/mL, but to show toxic effects at concentrations above 1000 ng/mL [4]. Falcarindiol (FaDOH), a bitter-tasting constituent in carrots [5], is reported to be part of the plant’s defence against fungal infections [6]. Falcarindiol 3-acetate (FaDOAc) has been described as more cytotoxic than FaDOH [7].

Falcarinol is reported to be the precursor to the two other FaTP, with FaDOH being the result of oxidation of FaOH, and FaDOAc being synthesised from FaDOH [8,9]. Both FaDOH and FaDOAc have been shown to be more abundant in the upper and outer parts of the root, while FaOH is more evenly distributed throughout the carrot root [10,11,12]. A more comprehensive description of FaTP in carrots can be found in [13].

Grown under field conditions, carrots exhibit considerable variation in FaTP concentrations depending on, e.g., size [14], the genotype used [10,14,15], stress arising from the growing conditions [16], pathogens [17,18,19,20] and processing [4,10,14,21,22,23,24,25,26].

In a study using samples from four different locations in Sweden, harvested repeatedly during three years, we previously reported seasonal variations in the concentrations of FaDOH, but also differences in the concentrations of FaTP, depending on the location of the cultivation [27]. Analyses of the same set of samples also indicated the existence of an equilibrium regulating the level of FaOH that is influenced by different external factors, such as location of carrot growth and storage conditions [28].

In another study, we examined the influence of different organic manures on FaTP concentrations in carrots [29]. The samples used were collected from a long-term field trial. The results, based on 288 carrot samples, indicated that differences in FaTP concentrations arising from manuring strategies were rather small and inconsistent [29].

In general, soil traits have been reported to influence both growth and quality in carrots [30,31]. Three major investigations have examined the effects of soil traits on FaTP concentrations in carrots. Lund and White [16] found that, compared with a control, carrots grown under either excess or deficiency of soil water exhibited decreasing concentrations of all three major FaTP. Czepa and Hoffmann [10] compared carrots grown in three different soils and, without describing the traits of the soils, concluded that differences in the cultivar used were more important for the composition of FaTP than soil type. Kramer et al. [32] conducted an experiment similar to that of Lund and White [16] and concluded that carrots grown either with low or high water supply exhibited different levels of FaTP than carrots with a moderate water supply, again with different cultivars responding differently to the same treatment.

As FaTP are reported to contribute important properties of the carrot root and also to be highly dependent on external factors, a better understanding of the correlation between soil conditions and polyacetylene concentrations is essential. Therefore, the present study investigated whether the chemical composition of the soil influences FaTP concentrations in carrots.

## 2. Materials and Methods

**Figure 1:**
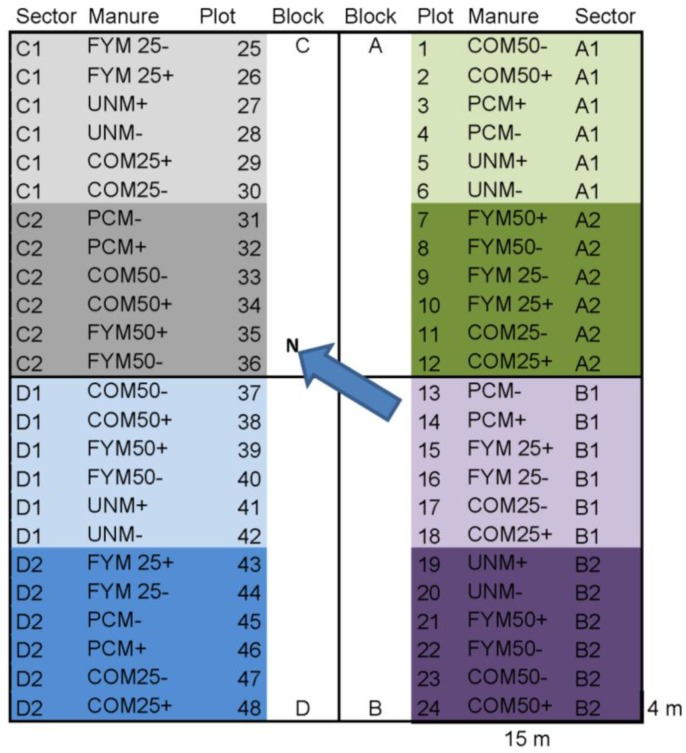
Design of field trial at Skilleby Research Farm 2006, coloured according to sectors used in data analysis. FYM = fresh farmyard manure, COM = composted farmyard manure, PCM = pelleted chicken manure, 25.50 t/ha, +/− = with/without biodynamic preparations.

Organic carrots, cv. Kämpe, an open-pollinated carrot of the Chantenay type, grown from seed obtained from Lindbloms Frö, Sweden, were harvested on three occasions (in total 144 samples) from a long-term field experiment at Skilleby research farm, Sweden (59.2° N, 17.4° E). The field experiment, described more in detail elsewhere [29], was established in 1991 to compare the influence of different organic manures on the properties of soils and crops. The design of the field trial is given in Figure 1.

Some of the carrot samples used were also analysed in our earlier study examining the influence of organic manures on carrot crops [29]. However, within the framework of that study it was not possible to make a more thorough analysis of the influence of soil chemical composition on FaTP concentrations in carrots.

The soil at the field trial site is mainly a clayey loam, with an organic carbon content of between 1.9% and 2.9%. The soil under the topsoil is stratified, with glacial layered clay at the bottom. The topsoil has undergone secondary sorting of the soil fractions (post-glacial clay, loam and silt) since the last ice age. The soil is generally high in potassium (K) and low in phosphorus (P) and has a pH between 5.7 and 6.5.

### 2.1. Sampling and Post-Harvest Treatment

Sampling for soil analysis was performed in all 48 plots of the field trial on 10 September 2006. Harvesting of carrots was performed in all 48 plots on three occasions during August and September 2006 (for harvesting dates see Table 1). Each carrot sample, consisting of 40–60 roots, was collected from 6–7 spots distributed all over each of the 48 field trial plots. The carrots were brought to the laboratory in cold storage boxes within 24 h. Weight, length, maximum diameter and mean thickness were determined on each root from all samples. After morphological analysis, the upper and lower ends of each carrot were removed and the remainder of the root was cut into cubes 0.5–1.0 cm in thickness. Approximately 60 g from each sample were frozen and kept at −80 °C. Before chemical analysis of FaTP and soluble sugars, all samples were freeze-dried and milled to a powder using an Ika-werke Yellow line type A10 mill from Staufen, Germany. Chemical analyses were performed directly after milling. Each sample was extracted in triplicate and each extraction was analysed separately.

### 2.2. Analysis

#### 2.2.1. Polyacetylenes in carrots

Samples were analysed for their FaTP content by HPLC using a 100 mm × 3 mm, particle size 3 µm, Luna C18 (2) column (Phenomenex, Torrance, CA, USA) at 40 °C, combined with an Agilent 1100 HPLC system equipped with a diode-array detector (Agilent Technology, Santa Clara, CA, USA) according to methods described elsewhere [1,33], with modifications described earlier [27]. The concentrations of FaTP were expressed as μg FaDOH equivalents/g DW by using an external certified standard of FaDOH from Atomax Chemicals Co. (Shenzhen, China). By dividing the linear constant of the internal standard, 4-chlorobenzophenone, at 205 nm by the corresponding constant for FaDOH at 205 nm, a factor of 1.235 was obtained and used to express the concentrations of polyacetylenes.

#### 2.2.2. Soluble Sugars in carrots

Analysis of fructose, glucose and sucrose was performed with a HPLC system (HPLC Technology Ltd., Welwyn Garden City, UK) equipped with an R14 IR detector and an Asahipac Shodex NH2P-50 4E column (4.6 mm × 250 mm; Showa Denko K.K., Tokyo, Japan) according to methods described elsewhere [28]. The concentrations of soluble sugars were expressed as mg/g DW.

#### 2.2.3. Soil

Soil samples from the upper soil layer (0–30 cm) of each of the 48 plots of the field trial were sent to Agrilab (Uppsala, Sweden), where they were analysed according to common standards: Total carbon (C) and nitrogen (N) content were measured with a LECO CHN 600 element analyser (SS-ISO 11464). Available P, K, calcium (Ca), magnesium (Mg) and sodium (Na) were analysed after extraction in ammonium lactate (AL) solution (SS 028310), total P, K, Mg, Ca and copper (Cu) were determined according to SS 028311 after extraction in hydrochloric acid (HCl) and pH was determined according to SS-ISO 10390.

### 2.3. Treatment of Data

The computer programs Excel 2010 (Microsoft Corp., Redmond, WA, USA), Minitab 16.0 (Minitab Inc., State College, PA, USA), SPSS 16.0 (IBM Inc., Armonk, NY, USA) and Simca 14.0 (MKS Data Analytics Solutions AB, Umeå, Sweden) were used for calculations and statistical evaluations. One-way ANOVA analysis together with the Duncan post hoc test at a significance level of *p* < 0.05 was used to determine differences between subjects. A bivariate correlation test was used to calculate the Pearson correlation coefficient. Principal component analysis (PCA) was used to describe multivariate interactions.

Data on temperature and precipitation, obtained from a weather station situated at the field trial, are given in Table 1.

## 3. Results

At the final harvest, mean root weight was 109 g, which is moderate. Mean total concentration of FaTP was 600 µg FaDOH equivalents/g dry weight (DW) and the mean contribution of the different FaTP to the total concentration was: FaDOH 76.2%, FaOH 16.2% and FaDOAc 7.6% (Table 1). This is in accordance with our earlier findings [29]. The concentrations of all three FaTP were higher at the first two harvests than at the harvest on 24 September (Table 1).

At the time of soil sampling (10 September), mean root weight was 66 g and mean concentration of FaTP was 701 µg FaDOH equivalents/g DW, with considerable variation between the samples (Table 1).

### 3.1. Soil Conditions within the Field Trial

**Figure 2:**
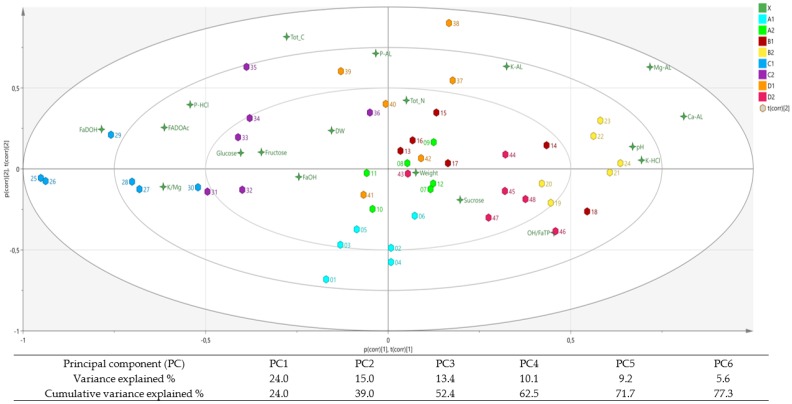
Biplot from principal component analysis (PCA) of soil and carrot variables at harvest on 10 September 2006. Diagram shows principal component (PC) scores for PC1 and PC2. Colours indicate variables and sectors of blocks in the field trial, numbers indicate plot number in the field trial (see Figure 1). Insert: Table of explained variance values for PCs 1–6.

The component scores from PCA on the variance of soil and root traits in the different parts of the field trial (see Figure 1) are shown in Figure 2. As can be seen, the biplot samples from block C were situated to the left and samples from blocks B were situated to the right along the first component (Figure 2). The component scores indicated presence of a gradient running diagonally across the field trial from the first plots in block C to the last plots in block B (Figure 2). Samples from block A were situated in the lower part and samples from block D were situated in the upper part of the second component (Figure 2). Within each block, samples from the six plots located on the edges of the field trial diverged in their component scores from samples from the six plots situated towards the middle of the trial (Figure 2). In the subsequent treatment of data, each block of the field trial was therefore divided into two equal parts, giving a total of eight sectors (Figure 1).

The results of the soil analysis showed that, in general, the soil could be categorised as low in pH and very low in available phosphorus, but high in available potassium and available magnesium (Table 2). Soil samples from sector A1 were low in pH, carbon, potassium, magnesium, calcium and total phosphorus (Table 2). Samples from sector B2 were high in pH, available phosphorus, magnesium, calcium and total potassium, but low in available potassium (Table 2). Samples from sector C1 were low in pH, potassium, available magnesium and calcium, but high in total phosphorus, K/Mg ratio and total potassium (Table 2). Soil samples from the fourth edge sector, D2, were low in carbon, nitrogen, phosphorus and copper (Table 2).

### 3.2. Soil Conditions, Polyacetylenes, Soluble Sugars and Carrot Size

There were significant differences between the sectors of the field trial in both FaTP concentrations and concentrations of soluble sugars, but not in carrot size, e.g., root weight (Table 3).

At the harvest on 10 September, the highest levels of all three FaTP were found in samples from sector C1 (Table 3). Higher levels of FaDOH and FaDOAc were also found in samples from sector C2 than in those from sectors A1 and A2 (Table 3). Carrots from sectors C1 and D2 exhibited low levels of sucrose than carrots from sectors A1, A2, B2 and D1 (Table 3). Samples from sectors C1 and D1 contained higher levels of fructose than samples from sector B1 and higher levels of glucose than samples from sectors A2, B1 and C2 (Table 3.)

At the harvest on 26 August, higher levels of FaDOH were found in samples from sectors C1 and C2 than in carrots from sector D2 (Table 3). Higher levels of FaDOAc were found in carrots from sector C2 than in carrots from sector A2 (Table 3). Higher levels of FaOH were found in samples from sector C2 compared with samples from sectors A1, B1, B2, D1 and D2 (Table 3).

At the harvest on 24 September, higher levels of FaDOH and FaDOAc were found in samples from sectors C1, C2, D1 and D2 than in samples from all other sectors (Table 3). Higher levels of FaOH were found in samples from sector D2 than in samples from sectors A1, A2, B1, B2 and C1 (Table 3). Samples from sector D2 also exhibited higher levels of fructose than samples from sectors A1, A2, C1 and D1.

### 3.3. Correlations between Soil Parameters

Among the soil samples, there was a positive correlation between pH, potassium, available magnesium and available calcium and a negative correlation between pH and total phosphorus (Table 4). The concentration of available phosphorus in the soil was positively correlated with the concentration of carbon or nitrogen (Table 4). There was also a positive correlation between available magnesium and total potassium (Table 4). The K/Mg ratio was negatively correlated with available calcium and with total concentration of potassium and copper (Table 4).

The biplot from the PCA analysis showed that the scores for available magnesium or calcium were situated to the right along the first component, together with the scores for total potassium and pH (Figure 2). According to the same PCA, the scores for total phosphorus and K/Mg ratio were situated more to the left along the first component, and the scores for carbon, nitrogen, available potassium and phosphorus more along the upper part of the second component (Figure 2).

### 3.4. Soil Parameters and Polyacetylenes

In the PCA biplot, the component scores for FaDOH and FaDOAc were situated more along the left side of the first component, opposite to the score for the FaOH/FaTP ratio, with the score for FaOH situated in between (Figure 2). The samples high in FaDOH showed component scores placing them more related to soil samples exhibiting high total phosphorus and/or high K/Mg ratio, but also opposite to soil samples high in pH, potassium and available calcium (Figure 2).

The total concentration of FaTP was positively correlated with the total concentration of phosphorus and the K/Mg ratio, but negatively correlated with the available magnesium or calcium concentration, total potassium concentration and pH (Table 4). The concentration of FaDOAc was positively correlated with the K/Mg ratio and total phosphorus concentration (Table 4). The concentration of FaOH only showed one weakly positive correlation, with the K/Mg ratio (Table 4). There was a negative correlation between the FaOH/FaTP ratio and the carbon concentration in the soil (Table 4).

Carrot samples grown in experimental site sectors low in total carbon exhibited a higher FaOH/FaTP ratio than samples grown in sectors with a higher total carbon concentration (although the number of samples was unevenly distributed between the PCA plot quartiles in this case) (Table 5). Samples grown in sectors with high levels of total phosphorus (>55 mg/100 g) exhibited higher concentrations of FaDOH or FaDOAc than samples grown in sectors with levels of total phosphorus of <51 mg/100 g soil (Table 5). Samples grown in plots rich in total potassium (>249 mg/100 g) exhibited lower levels of FaDOH than samples grown in plots with total potassium concentrations <230 mg/100 g soil (Table 5). The concentrations of FaDOH and FaDOAc increased with increasing K/Mg ratio (Table 5). The level of nitrogen in the soil did not have a significant influence on the concentrations of FaTP in the carrots, which were also not influenced by the level of available phosphorus or the level of available potassium (data not shown).

## 4. Discussion

This study showed that the chemical composition of the soil influenced the concentrations of FaTP in carrots. However, the size and shape of the carrots was not influenced by the differences in soil traits. Therefore, the reason for the differences in FaTP concentrations must instead lie in differences in root physiology between the carrot samples.

The analysis revealed a strong positive correlation between the concentrations of FaDOH in carrots and the total concentration of phosphorus in the soil. Phosphorus is an important plant macronutrient, making up about 0.2% of a plant’s dry weight. It is a component of key molecules such as nucleic acids, phospholipids and ATP [34]. Compared with, e.g., cabbage, carrots have been reported to possess weaker phosphorus use efficiency [35]. The level of readily available phosphorus in the field trial was low, probably causing a general risk of phosphorus deficiency in the carrots. In combination, the high levels of total phosphorus in soil and low levels of available phosphorus are a strong indication that the carrots in the study could not solubilise sufficient phosphorus. As the differences between samples in terms of available phosphorus were small, it was the levels of total phosphorus that apparently had a significant impact on the concentrations of FaDOH and FaDOAc. To investigate this further, studies on soils with a broader spectrum of readily available phosphorus levels are required.

In soils low in available phosphorus, plants must rely more on soil bacteria and arbuscular mycorrhizal fungi for phosphorus solubilisation [36,37]. This symbiosis is particularly important in organic cropping, where limited amounts of easy soluble phosphorus are applied [38,39,40]. The concentrations of FaTP in carrots are reported to increase in the presence of fungi [6,17,41,42]. The positive correlation observed in the present study between the concentrations of FaDOH or FaDOAc in carrots and the levels of total phosphorus in soil might also be explained by higher amounts of mycorrhiza surrounding the roots. To confirm this, the concentrations of FaTP concentrations in carrots grown in soils where the degree of mycorrhizal infection is known need to be determined.

The concentration of FaDOH and the total concentrations of FaTP exhibited a negative correlation with the concentrations of readily available magnesium and calcium. This indicates that the concentrations of FaDOH in carrots may be higher when they are grown in soils poor in available minerals. Low pH levels often make it more difficult for the crop to take up some minerals from the soil. The negative correlation observed here between the concentrations of FaTP and pH level also points in the same direction; that synthesis of FaDOH is stimulated in carrots growing under nutrient stress.

Earlier studies have reported that the levels of FaTP in carrots are correlated with external factors that stress the carrot plant during its growth [16,32]. The concentrations of FaDOH have been shown to be influenced most, and the concentrations of FaOH least, by external factors [10,13,14,32]. The biosynthesis of FaTP begins with FaOH [9]. When stress occurs in carrots, it is likely that the level of FaOH increases initially. Depending on root size, and probably also on the degree of stress, the transformation of FaOH to FaDOH and to FaDOAc proceeds at different rates. Under stress, the total concentrations of the three major FaTP should therefore increase, and together give a good indication of the stress level. However, Lund and White [43] found the opposite: In carrots stressed by drought neither FaDOH nor FaOH could be found, but both were present in the control. Furthermore, the concentrations of FaDOAc and of five other FaTP sometimes present in carrots increased in the carrots stressed by drought [43]. Recent studies have mainly focused on the three major FaTP, but a broader analysis involving all FaTP occurring in carrots is perhaps needed to increase understanding of the dynamics of FaTP in carrots.

In an earlier paper we discussed the influence of organic manures on FaTP concentrations in carrots [29]. The samples used in that study came from the same field trial as the samples used here. The field trial provided large variation in both the type and amounts of manure applied. In comparison with the differences reported here, the differences in the concentration of FaTP between different manuring strategies were small [29]. At the harvest on 10 September there were no differences in FaTP concentrations between the manure treatments. The rather small differences in soil chemical composition reported here indicate that the concentrations of FaTP in carrots are much more dependent on the prevailing soil conditions than on manuring strategies.

Sucrose is the main sugar used in transportation from leaf to root in the plant [44] and its decomposition to hexoses in the root is reported to be enzymatic [45,46,47] and dependent on the respiratory rate [48,49]. The results presented here support the assumption made in our earlier study [28] concerning a possible positive correlation between the concentrations of FaTP and the respiratory rate. They also support the assumption that the enzyme transforming FaOH to FaDOH might be active under similar conditions as the enzymes transforming sucrose to hexoses [28]. However, this needs to be confirmed in further experiments by directly measuring enzymatic activity and respiratory rate.

Regarding the FaTP concentrations, the samples from the field trial were analysed block by block and high concentrations of FaDOH were found mainly in one of the blocks. It could be argued that the differences were due to analytical error, but under deeper consideration this possibility was rejected for three main reasons: (i) The variation in the internal standard did not differ between the batches; (ii) the concentrations of FaDOH decreased consistently, from field plot to field plot in the block with the high concentrations and were not correlated with decreased concentrations of e.g., FaOH; and (iii) high concentrations of FaDOH were found in the block in question at the harvests before and after that on 10 September. Therefore, it does not seem likely that analytical error influenced the results in a crucial way.

Although the analyses comparing plant and soil concentrations of FaTP were based on 48 different samples, these were taken on only one harvest occasion. This is not enough to allow more consistent assumptions to be drawn, particularly since the variation in soil conditions between samples was rather small. Adding the results from the two adjacent harvests in 2006 strengthened the assumptions drawn in this study, but in order to increase the significance of the results, experiments on soils having a broader variation in chemical composition are needed. Due to the reported influence of growing conditions on FaTP concentrations [4,10,14,27], it is important that these experiments are performed at the same location.

Positive effects on human health have been attributed to FaOH [2], whereas FaDOH has been described as contributing to the bitter off-taste in carrots [5]. A high ratio between FaOH and total FaTP can therefore be taken as a sign of better composition of the FaTP in carrots. In the PCA performed in this study, the component scores for the FaOH/FaTP ratio were situated close to those for the concentrations of sucrose and opposite those for the concentrations of FaDOH, FaDOAc and hexoses, indicating that the FaOH/FaTP ratio increases as carrots develop. This is in agreement with earlier findings [29], and indicates that late harvesting might be preferable, at least if the external circumstances are normal.

The results from the PCA were confirmed results obtained by more conventional statistical methods. The total variance explained in the PCA used was somewhat low. Despite this, the PCA arranged the component scores from the different plots more or less in accordance with their position in the field trial. The PCA also revealed a negative correlation between pH and FADOH that could not be determined by one-way ANOVA and that was only vaguely indicated when using bivariate correlation analysis.

The concentrations of soluble sugars were also influenced by the chemical composition of the soil. The ratio between sucrose and hexoses normally increases during the growing season and has been proposed as an indicator of carrot root development [44,45,50]. At the harvest on 10 September, there was a strong negative correlation between the concentration of FaDOH and both the concentration of sucrose (*p* = −0.403 **) and the sucrose/hexose ratio (*p* = −0.384 **). This indicates that the carrots high in FaDOH at this time were slightly delayed in their physiological development compared with carrots lower in FaDOH.

The results showed a strong negative correlation between FaOH/FaTP ratio and the carbon concentration in the soil. The PCA also showed a positive correlation between total carbon and total nitrogen, easily available phosphorus and potassium. This points toward a soil more rich in nutrients. The score for total carbon was also correlated with the scores for the two hexoses along the first principal component. Taken together, it appears that carrots grown in a soil richer in carbon and nutrients were delayed in their development. In order to examine this relationship more closely, however, further experiments with a wider variation in the levels of carbon in the soil are required.

We previously reported that the concentrations of FaTP in carrots exhibit a seasonal rhythm, regardless of root size [27]. Here, the concentration of each individual FaTP was significantly lower at the third harvest than at the two preceding harvests. It is possible the differences in soil conditions caused this variation in carrot development. The higher concentrations of FaTP in carrots grown in some soils would in that case be primarily attributable to a delay in the physiological development of the carrots, with only a secondary dependence on soil traits. The differences in FaTP concentrations observed here may have partly been the result of delayed physiological development in carrots grown in poor soil. However, the differences persisted during the latter part of the growing season, when no signs of delayed physiological development were noticed. Thus higher concentrations of FaTP may also be an outcome of interactions between the carrot root surface and its surrounding environment.

The concentrations of FaDOH in carrots have been reported to decrease as the size of the carrot root increases [14]. It might be argued that the differences between samples reported here were caused by differences in root size, rather than the actual soil conditions, if the carrots grown in poor soil were smaller than those grown in richer soil. However, there was no consistent difference in root size among the carrot samples compared, so this explanation does not apply here.

The total concentrations of FaTP and of its major constituent, FaDOH, were correlated to the levels of potassium, phosphorus and magnesium in the soil. The soil in which the carrot samples were grown was rich in available potassium and available magnesium, but the K/Mg ratio was low. A low K/Mg ratio is generally considered to decrease the potential of the carrot crop to assimilate potassium. Our results showed that the concentration of magnesium had a stronger impact than the concentration of potassium on the levels of FaDOH. The significant positive correlation between the K/Mg ratio and FaDOH was thus probably influenced more by magnesium than by potassium. If high levels of FaDOH are a sign of stress in carrots, high levels of magnesium in relation to potassium seem essential for optimal development.

Overall, the results show that the levels and composition of FaTP in carrots are influenced by the chemical conditions in the soil. The effects are probably caused not only by the ratio between total phosphorus and potassium, but also by the pH level and by the levels of other minerals, such as calcium and magnesium. The relatively small variation between the soil samples, together with the complexity of soil dynamics, makes further investigations necessary in order to increase understanding of the relationship between soil and FaTP in carrots. 

## 5. Conclusions

A novel finding in this study was that the chemical composition of the soil influences the concentrations of FaTP in carrots. Carrots grown in soil generally low in available phosphorus had higher levels of FaDOH if the soil was also low in available magnesium and calcium. The FaTP concentrations were positively correlated with the total level of phosphorus in the soil, but negatively correlated with the total level of soil potassium. Of the three FaTP analysed in carrots, FaDOH concentrations were influenced most by changes in soil chemical composition, with high levels of FaDOH more likely to occur when the soil conditions were poor.

## Figures and Tables

**Table 1:** Sampling date, sum of precipitation and temperature at the time of sampling and mean traits of carrot samples harvested on three different occasions in 2006. Means within the same row followed by different letters are significantly different according to one-way ANOVA with Duncan’s post-hoc test (*p* < 0.05). *n* = 48.

Harvest Date	26 August	10 September	24 September
Harvest number	1	2	3
Cultivation days	104	119	133
Precipitation, mm ^1^	153	154	175
Degree-days °C ^1^	1874	2098	2277
Weight, g	30 ± 8 c	66 ± 18 b	109 ± 22 a
FaDOH, μg/g DW ^2^	503 ± 87 a	529 ± 203 a	340 ± 178 b
FaDOAc, μg/g DW ^2^	50 ± 13 a	54 ± 27 a	32 ± 24 b
FaOH, μg/g DW ^2^	104 ± 29 a	118 ± 41 a	69 ± 58 b

^1^ Sum of precipitation and sum of mean daily temperature 2006, from 1 April until harvest; ^2^ Quantified as FaDOH equivalents.

**Table 2:** Mean soil parameters in each of the eight sectors (A1–D2) within the field trial. Sampling date 10 September 2006. Means within the same column followed by different letters are significantly different according to one-way ANOVA with Duncan’s post hoc test (*p* < 0.05).

Sector	*N*	pH	Tot C%	Tot N%	P (Al) mg/100 g	P (HCl) mg/100 g	K (Al) mg/100 g	K (HCl) mg/100 g	Mg (Al) mg/100 g	K (Al)/Mg (Al)	Ca (Al) mg/100 g	Cu (HCl) mg/1000 g
A1	6	5.9 ± 0.1 cd	2.05 ± 0.1 d	0.25 ± 0.02 ab	2.0 ± 0.3 bc	47.8 ± 2 b	13.2 ± 1 d	230 ± 12 cd	22.1 ± 3 e	0.60 ± 0.09 bc	197 ± 13 e	17.2 ± 2 bc
A2	6	5.8 ± 0.1 d	2.35 ± 0.2 abc	0.27 ± 0.02 ab	2.3 ± 0.4 abc	52.3 ± 3 a	14.6 ± 1 cd	254 ± 14 b	26.6 ± 1 cd	0.54 ± 0.02 c	213 ± 6 d	20.2 ± 1 a
B1	6	6.0 ± 0.1 c	2.43 ± 0.2 ab	0.28 ± 0.03 a	2.1 ± 0.4 abc	54.7 ± 2 a	14.6 ± 1 cd	285 ± 11 a	29.9 ± 2 ab	0.49 ± 0.02 d	236 ± 8 c	20.2 ± 1 a
B2	6	6.3 ± 0.2 a	2.15 ± 021 cd	0.28 ± 0.06 a	2.4 ± 0.6 a	47.1 ± 2 b	16.1 ± 1 bc	301 ± 16 a	29.3 ± 1 abc	0.55 ± 0.03 bc	289 ± 14 a	18.4 ± 3 abc
C1	6	5.9 ± 0.1 cd	2.33 ± 0.1 bc	0.25 ± 0.03 ab	2.2 ± 0.2 abc	55.3 ± 4 a	14.5 ± 2 cd	227 ± 11 cd	19.3 ± 2 e	0.75 ± 0.07 a	193 ± 11 e	17.8 ± 1 bc
C2	6	5.8 ± 0.1 d	2.55 ± 0.2 ab	0.28 ± 0.02 a	2.5 ± 0.4 abc	53.5 ± 4 a	14.8 ± 3 cd	219 ± 16 d	25.6 ± 4 d	0.58 ± 0.06 bc	214 ± 13 d	18.9 ± 2 ab
D1	6	6.0 ± 0.1 c	2.57 ± 0.3 a	0.27 ± 0.03 ab	2.7 ± 0.7 ab	51.5 ± 5 ab	18.4 ± 2 a	246 ± 24 bc	31.3 ± 4 a	0.59 ± 0.04 bc	242 ± 17 c	19.1 ± 2 ab
D2	6	6.1 ± 0.1 b	2.05 ± 0.1 d	0.24 ± 0.02 b	1.7 ± 0.3 c	42.0 ± 5 c	17.2 ± 1 ab	264 ± 23 b	27.9 ± 1 bcd	0.62 ± 0.04 b	267 ± 11 b	16.7 ± 1 c
Total	48	6.0 ± 0.2	2.3 ± 0.3	0.27 ± 0.03	2.3 ± 0.6	50.5 ± 5	15.4 ± 2	253 ± 31	26.5 ± 4	0.59 ± 0.09	253 ± 31	18.6 ± 2

**Table 3:** Concentrations of FaTP, concentrations of soluble sugars and root weight in carrot samples from different sectors of the field trial at the three harvests in 2006. Means within the same section of a column followed by different letters are significantly different according to one-way ANOVA with Duncan’s post hoc test (*p* < 0.05).

Harvest	Sector	FaDOH μg/g DW ^1^	FaDOAc μg/g DW ^1^	FaOH μg/g DW ^1^	FaOH/FaTP%	Fructose mg/g DW	Glucose mg/g DW	Sucrose mg/g DW	Weight g
26 August	A1	528 ab	44 ab	91 cd	13.8 bc	194 a	237 a	61 a	33.5 a
	A2	483 ab	41 b	113 abc	17.9 ab	171 ab	206 ab	72 a	33.1 a
	B1	473 ab	44 ab	81 d	13.9 bc	147 bc	175 bc	39 b	30.7 a
	B2	494 ab	47 ab	78 d	12.7 c	136 c	170 bc	41 b	25.7 a
	C1	575 a	55 ab	126 ab	16.8 abc	129 c	164 c	41 b	28.7 a
	C2	552 a	59 a	140 a	19.4 a	123 c	151 c	44 b	32.3 a
	D1	479 ab	52 ab	99 bcd	15.7 abc	121 c	150 c	36 b	30.0 a
	D2	439 b	57 ab	98 cd	16.5 abc	117 c	142 c	34 b	28.1 a
10 September	A1	362 d	36 c	113 abc	22.5 ab	155 ab	190 ab	162 a	63.3 a
	A2	381 cd	31 c	84 c	16.8 bc	144 ab	176 b	150 a	75.4 a
	B1	446 cd	51 bc	99 bc	16.9 bc	133 b	160 b	132 ab	58.8 a
	B2	420 cd	48 bc	140 ab	22.9 a	147 ab	183 ab	151 a	67.0 a
	C1	898 a	95 a	155 a	13.5 c	174 a	219 a	104 b	59.7 a
	C2	760 b	71 b	129 abc	13.5 c	144 ab	179 b	131 ab	68.5 a
	D1	493 c	52 bc	107 abc	16.3 c	176 a	218 a	155 a	65.6 a
	D2	470 cd	48 bc	120 abc	18.3 abc	154 ab	188 ab	108 b	66.8 a
24 September	A1	129 b	7 b	10 c	6.8 c	139 b	163 a	166 a	106.1 a
	A2	181 b	11 b	16 c	8.0 c	139 b	159 a	159 a	116.4 a
	B1	198 b	14 b	23 c	9.5 c	145 ab	169 a	139 abc	111.2 a
	B2	237 b	15 b	30 c	10.6 c	142 ab	169 a	124 bc	107.1 a
	C1	547 a	53 a	107 b	15.2 b	136 b	161 a	122 c	106.2 a
	C2	484 a	47 a	111 ab	17.0 ab	141 ab	161 a	158 ab	119.6 a
	D1	465 a	49 a	110 ab	18.1 ab	139 b	166 a	160 a	104.7 a
	D2	477 a	58 a	145 a	21.3 a	152 a	170 a	139 abc	97.8 a

^1^ Quantified as FaDOH equivalents.

**Table 4:** Correlation matrix; soil and carrot samples from harvest 2 (10 September). Pearson correlation coefficients coloured according to significance level; *n* = 48.

	pH	P (Al)	K (Al)	Mg (Al)	K/Mg	Ca (Al)	K (HCl)	P (HCl)	Cu (HCl)	Tot C	Tot N	FaDOH	FADOAc	FaOH	FaTP	FaOH/FaTP
pH	1															
P (Al)	0.19	1														
K (Al)	0.43 **	0.34 *	1													
Mg (Al)	0.48 **	0.30 *	0.67 **	1												
K/Mg	−0.15	0.00	0.18	−0.61 **	1											
Ca (Al)	0.82 **	0.24	0.56 **	0.75 **	−0.38 **	1										
K (HCl)	0.60 **	−0.10	0.21	0.51 **	−0.41 **	0.65 **	1									
P (HCl)	−0.51 **	0.14	−0.23	−0.14	0.01	−0.47 **	−0.07	1								
Cu (HCl)	−0.26	0.05	0.06	0.35 *	−0.38 **	−0.05	0.31 *	0.61 **	1							
Tot C	−.030 *	0.47 **	0.28	0.34 *	−0.10	−0.10	−0.23	0.62 **	0.46 **	1						
Tot N	−0.08	0.49 **	0.07	0.21	−0.18	0.06	0.03	0.24	0.14	0.45 **	1					
FaDOH	−0.26	0.04	−0.07	−0.42 **	0.50 **	−0.37 *	−0.40 **	0.44 **	−0.06	0.22	−0.02	1				
FADOAc	−0.15	0.12	0.01	−0.29 *	0.44 **	−0.22	−0.22	0.38 **	−0.05	0.16	−0.01	0.84 **	1			
FaOH	0.07	0.15	0.01	−0.24	0.35 *	−0.02	−0.02	0.08	−0.12	−0.14	0.12	0.45 **	0.45 **	1		
FaTP	−0.33 *	0.06	−0.14	−0.52 **	0.55 **	−0.47 **	−0.44 **	0.42 **	−0.09	0.14	0.01	0.97 **	0.84 **	0.57 **	1	
FaOH/FaTP	0.24	0.14	−0.03	0.03	−0.08	0.23	0.26	−0.36 *	−0.11	−0.42 **	0.07	−0.52 **	−0.37 **	0.48 **	−0.35 *	1

** Positive correlation significant at *p* < 0.01 (two-tailed) coloured in dark red; * Positive correlation significant at *p* < 0.05 (two-tailed) coloured in light red; ** Negative correlation significant at *p* < 0.01 (two-tailed) coloured in dark blue; * Negative correlation significant at *p* < 0.05 (two-tailed) coloured in light blue.

**Table 5:** Quartiles of soil parameters in relation to FaTP concentrations and root weight at harvest on 10 September. *n* = 48. Means within the same section of a column followed by different letters are significantly different according to one-way ANOVA with Duncan’s post hoc test (*p* < 0.05).

Soil trait	Quartiles		FaDOH μg/g DW ^1^	FaDOAc μg/g DW ^1^	FaOH μg/g DW ^1^	Total FaTP μg/g DW ^1^	FaOH/FaTP%	Root weight, g
Total carbon %	1.9–2.1	17	464 a	50 ab	120 a	634 a	19.8 a	61 a
	2.2–2.3	10	491 a	41 b	113 a	645 a	18.2 ab	70 a
	2.4–2.5	12	608 a	65 a	129 a	803 a	16.5 ab	66 a
	2.6–2.9	9	586 a	62 ab	106 a	754 a	14.2 b	68 a
Phosphorus (HCl) mg/100 g	37–47	12	449 b	50 ab	124 a	622 b	20.1 a	65 a
	47–51	12	447 b	37 b	114 a	598 b	19.1 ab	65 a
	51–55	13	583 ab	64 a	111 a	758 ab	16.0 ab	68 a
	55–59	11	640 a	66 a	126 a	832 a	15.0 b	65 a
Potassium (HCl) mg/100 g	198–230	12	632 a	62 a	115 a	809 a	15.9 a	66 a
	230–248	13	584 ab	60 a	131 a	774 ab	17.3 a	69 a
	249–278	11	442 b	45 a	101 a	588 b	17.4 a	62 a
	279–314	12	445 b	49 a	124 a	618 ab	19.8 a	64 a
Potassium (Al)/ Magnesium (Al), %	46–53	12	456 b	48 b	104 a	607 b	17.4 a	64 a
	53–57	12	494 ab	48 b	113 a	654 b	17.4 a	69 a
	57–63	13	520 ab	51 ab	120 a	622 ab	18.2 a	64 a
	63–84	11	656 a	71 a	137 a	865 a	17.3 a	67 a

^1^ Quantified as FaDOH equivalents.

## Abbreviations

The following abbreviations are used in this manuscript:

FaDOH: falcarindiol
FaDOAc: falcarindiol-3-acetate
FaOH: falcarinol
FaTP: falcarinol-type polyacetylenes
P (Al): available phosphorus
K (Al): available potassium
Mg (Al): available magnesium
Ca (Al): available calcium
K (HCl): total potassium
P (HCl): total phosphorus
Cu (HCl): total copper
PCA: principal component analysis.
